# Supplementary material for: Identification of expressed endogenous retroviral element associated long terminal repeats in devil facial tumour cells
Source: Open Biol. 2026 Oct 7;16(10):250430. doi: 10.1098/rsob.250430 (PMC13641905; doi:10.1098/rsob.250430)
Supplement: Supplementary file 1 [file rsob.250430.zip › rsob.250430/rsob250430_si_003.pdf]

Supplementary Materials

Identification of Expressed Endogenous Retroviral Element Associated Long Terminal Repeats in Devil Facial Tumour Cells

Authors

Anuk Kruawan[1], Maximilian Stammnitz[2], Richard Wilson[3], Sri H. Ramarathinam[4], Chrissie E. B. Ong[1], Kirsten A. Fairfax[5], Amanda L. Patchett[6], A. Bruce Lyons[5], Andrew S. Flies[1]*

Affiliations

[1]Menzies Institute for Medical Research, College of Health and Medicine, University of Tasmania, Hobart, TAS 7000, Australia

[2]Centre for Genomic Regulation, the Barcelona Institute for Science and Technology (BIST), 08003 Barcelona, Spain

[3]Central Science Laboratory, University of Tasmania, Hobart, TAS 7001, Australia

[4]Department of Biochemistry and Molecular Biology and the Infection and Immunity Program, Biomedicine Discovery Institute, Monash University, Clayton, Victoria 3800, Australia

[5]School of Medicine, College of Health and Medicine, University of Tasmania, Hobart, TAS 7000, Australia

[6]Aquaculture Health Team, Agriculture & Food, Commonwealth Scientific and Industrial Research Organisation, Battery Point, TAS 7004, Australia

Corresponding author contact information

*Correspondence: andy.flies@utas.edu.au (A.S. Flies)

Menzies Institute for Medical Research, University of Tasmania

GPO Box 341

Hobart, Tasmania, 7001, Australiat: +61 3 6226 4614

Supplementary Table S2 Repeat elements identified in Tasmanian devils genome

The dataset has been doposited on https://doi.org/10.5281/zenodo.17282419. The dataset contains a list of repeat elements identified in the Tasmanian devil (*Sarcophilus harrisii*) genome (mSarHar1.11). For further details, please see https://doi.org/10.1126/science.abq6453. The dataset was generated using RepeatMasker v4.0.8 in sensitive mode, with Blastp version 2.0MP-WashU, utilising a combined database consisting of Dfam_Consensus-20181026 and RepBase-20181026. The following parameters were used: RepeatMasker -engine wublast -species 'sarcophilus harrisii' -s -no_is -cutoff 255 -frag 20000.
