# Supplementary material for: Identification of expressed endogenous retroviral element associated long terminal repeats in devil facial tumour cells
Source: Open Biol. 2026 Oct 7;16(10):250430. doi: 10.1098/rsob.250430 (PMC13641905; doi:10.1098/rsob.250430)
Supplement: Supplementary file 1 [file rsob.250430.zip › rsob.250430/rsob250430_si_004.pdf]

Supplementary Materials

Identification of Expressed Endogenous Retroviral Element Associated Long Terminal Repeats in Devil Facial Tumour Cells

Authors

Anuk Kruawan[1], Maximilian Stammnitz[2], Richard Wilson[3], Sri H. Ramarathinam[4], Chrissie E. B. Ong[1], Kirsten A. Fairfax[5], Amanda L. Patchett[6], A. Bruce Lyons[5], Andrew S. Flies[1]*

Affiliations

[1]Menzies Institute for Medical Research, College of Health and Medicine, University of Tasmania, Hobart, TAS 7000, Australia

[2]Centre for Genomic Regulation, the Barcelona Institute for Science and Technology (BIST), 08003 Barcelona, Spain

[3]Central Science Laboratory, University of Tasmania, Hobart, TAS 7001, Australia

[4]Department of Biochemistry and Molecular Biology and the Infection and Immunity Program, Biomedicine Discovery Institute, Monash University, Clayton, Victoria 3800, Australia

[5]School of Medicine, College of Health and Medicine, University of Tasmania, Hobart, TAS 7000, Australia

[6]Aquaculture Health Team, Agriculture & Food, Commonwealth Scientific and Industrial Research Organisation, Battery Point, TAS 7004, Australia

Corresponding author contact information

*Correspondence: andy.flies@utas.edu.au (A.S. Flies)

Menzies Institute for Medical Research, University of Tasmania

GPO Box 341

Hobart, Tasmania, 7001, Australiat: +61 3 6226 4614

Supplementary Table S3 List of normalised read counts for Tasmanian devil transcriptomes

The dataset has been doposited on https://doi.org/10.5281/zenodo.20192864 The dataset contains normalised read counts, TPM, and log2-transformed TPM for all long terminal repeats (LTRs) and canonical genes from the Tasmanian devil's whole-genome sequence, mSarHar1.11.

We generated the dataset by aligning RNA-seq datasets to mSarHar1.11 using STAR aligner for single-end and paired-end reads, according to the software manual. Technical replicates were combined using STAR. Subread featureCounts was used to obtain raw read counts. Normalisation was conducted using a non-linear full quantile normalisation method as part of EDAseq package. They were then converted to transcripts per kilobase million (TPM) and log2TPM using Scater.
